# Supplementary material for: Mixed reality simulation of guided needle insertion: effects of interaction and visual fidelity
Source: Int J Comput Assist Radiol Surg. 2026 Aug 10;21(11):2317–25. doi: 10.1007/s11548-026-03756-3 (PMC13759450; doi:10.1007/s11548-026-03756-3)
Supplement: Supplementary file 1 [file 11548_2026_3756_MOESM1_ESM.pdf]

Mixed Reality Simulation of Guided Needle Insertion: Effects of Interaction and Visual Fidelity

Florian Heinrich[1*], Marvin Kohpeiß[1], Christian Hansen[1], Danny Schott[1]

[1]Research Campus Stimulate, University of Magdeburg, Magdeburg, 39106, Saxony-Anhalt, Germany.

*Corresponding author(s). E-mail(s): florian.heinrich@isg.cs.uni-magdeburg.de; Contributing authors: marvin.kohpeiss@st.ovgu.de; hansen@isg.cs.uni-magdeburg.de; danny.schott@ovgu.de;

Abstract

Purpose: Mixed Reality (MR) simulations are increasingly used in medical training to provide safe, controllable, and repeatable practice environments. While visual fidelity (VF) and interaction fidelity (IF) are known to influence user experience (UX), their respective effects on performance and UX in complex, precision-based medical procedures remain insufficiently understood.

Methods: We conducted a within-subjects user study investigating the effects of VF (high vs. low) and IF (tangible vs. virtual interaction) in a precise needle insertion task. A non-immersive setup served as a reference condition. Performance metrics, perceived realism, workload, and UX were assessed across conditions.

Results: Both fidelity factors successfully modulated perceived realism. Tangible interaction significantly improved insertion depth control, reduced workload, and enhanced UX. High VF primarily increased spatial presence and involvement, with only limited benefits for task performance. Interaction effects revealed that tangible interaction provided greater advantages under low VF, particularly in terms of task completion time and needle alignment.

Conclusion: Visual and interaction fidelity play distinct and complementary roles in MR-based medical training. Tangible interaction predominantly supports procedural accuracy and efficiency, whereas high visual fidelity mainly enhances immersive engagement. MR system design should therefore align fidelity decisions with specific training objectives.

Keywords: Medical XR, Medical Task Simulation, Fidelity, Haptic Interaction

1 Introduction

Mixed Reality (MR) is increasingly used in medical education and system prototyping, as it enables controlled, repeatable, and ethically acceptable training and evaluation scenarios [1]. Its effectiveness has been demonstrated across surgical and procedural domains, where MR-based applications can support skill acquisition and learning [2]. However, purely virtual MR systems often lack realistic haptic feedback, which can limit usability and performance in precision-critical, tool-based tasks. To address this limitation, hybrid simulation approaches combine physical mock-ups with immersive virtual environments, allowing users to interact with tangible instruments while retaining the flexibility and controllability of MR [3, 4].

A key open question is how realistic immersive virtual environments (IVEs) must be for effective medical training. Fidelity is commonly understood as the degree to which an IVE reproduces real-world conditions through multisensory stimuli [5]. Prior work indicates that multisensory setups, including haptics, can improve user experience (UX) and presence, i.e., the sense of being there [6–9]. In this work, we treat fidelity as a multidimensional design property rather than as a single level of realism. Following Bonfert et al.'s [10] Interaction Fidelity Model, fidelity can be understood as the degree of correspondence between a simulated interaction and a defined reference interaction, with realism representing the special case in

which this reference is the real world. The model distinguishes multiple components across the human–computer interaction loop, including action, detection, transfer, simulation, rendering, display, perceptual, and experiential fidelity. Against this background, we use *visual fidelity* (VF) to refer primarily to the presentational, rendering, and visually perceived aspects of the simulated environment, including the realism and task-relevant detail of the environment, instruments, and anatomy. We use *interaction fidelity* (IF) to refer primarily to the action-related correspondence between the simulated task and its reference interaction, including how users' actions are performed, detected, mapped to virtual actions, and reflected in the system response. Thus, VF primarily concerns what users perceive visually, whereas IF in this study concerns how closely users can act within the simulation in correspondence with the reference interaction. Reviews emphasize that fidelity can positively influence performance, perception, UX, and presence, but effects are not uniform and depend on the fidelity dimension, task complexity, and user context [5, 11]. In learning settings, fidelity has been identified as a predictor of engagement, active learning behavior, and cognitive gains [12].

These findings underscore that fidelity should not be treated as a unitary construct, but that its effects need to be considered with respect to specific fidelity dimensions and task demands. To date, however, a substantial portion of the literature has focused on visual aspects only. Higher VF is consistently associated with increased presence, involvement, and perceived realism [8, 9]. However, visually simplified environments may be sufficient for low-precision tasks such as situated memory recall or exposure therapy, with limited impact on objective performance [13, 14]. Interaction-related aspects have also been studied: tangible mock-ups can improve task performance in usability testing [4], and congruence between VF and IF has been linked to performance benefits in VR tasks [15]. Dedicated interaction techniques for high-precision 3D manipulation further highlight the importance of interaction design for demanding tasks [16]. Nevertheless, much of this evidence stems from scenarios with comparatively low precision demands, and transferability to precision-critical medical procedures remains uncertain [17].

Within medical MR, several studies report that increasing VF improves presence and UX but yields limited or inconsistent gains in procedural performance [3]. Moreover, fidelity-related design choices are often insufficiently documented, complicating interpretation, comparison, and replication [18]. Consequently, whether and how VF and IF contribute to measurable performance benefits in demanding MR-based medical tasks remains an open question [3, 15, 16, 19].

To address this gap, we conducted a controlled within-subjects study examining how VF and IF influence objective performance and subjective experience in a precision-critical MR task. Using a guided needle insertion scenario representative of image-guided and minimally invasive procedures, we independently manipulated VF and IF in a 2×2 factorial design and included a non-immersive physical baseline for reference. VF was operationalized by comparing a visually detailed MR environment (high VF) with a visually simplified representation of the same task scenario (low VF). IF was operationalized by comparing tangible tracked instruments, which preserved the physical handling characteristics of the procedure, with a purely virtual interaction condition in which the task was performed without the corresponding physical objects. By quantifying effects on procedural accuracy, task efficiency, workload, UX, and presence, this work provides empirical evidence on the distinct and complementary roles of VF and IF in medical MR simulation. Rather than introducing a new interaction technique, the contribution lies in the controlled comparison of these fidelity dimensions and their implications for the design and evaluation of MR-based simulation and prototyping systems for computer-assisted radiology and surgery.

2 Materials

2.1 Simulation Task and Design

To support a systematic investigation of VF and IF in MR, we designed a medical simulation centered on a precision-critical needle insertion task. Rather than reproducing a complete clinical workflow, the simulation focuses on selected procedural elements that require tool-based interaction and spatial coordination. This design choice was motivated by prior work reporting limited performance effects of fidelity manipulations in low-precision medical tasks, indicating the need to examine more demanding interactions [3]. The simulation is based on a clinically inspired workflow and comprises sequential sub-tasks with increasing precision requirements.

- **(a) Placement of medical instruments:** The user moves an instrument table from an initial position to the interventional workspace, encouraging gross motor interaction and spatial exploration of the IVE.
- **(b) Sterilization of the puncture site:** The user disinfects a laser-marked area on the patient surface using controlled hand movements, supporting surface-based interaction and spatial coordination.
- **(c) Needle insertion:** The user inserts a needle toward a predefined anatomical target, requiring accurate control of insertion angle and depth.

Sub-tasks (a) and (b) are low in precision demands and primarily serve to familiarize participants with the environment, tools, and spatial layout of the IVE. The analysis of task performance therefore focuses on sub-task (c), which captures the precision-critical aspects of the procedure. This progressive task structure follows established practices in simulation-based training and supports skill acquisition and confidence building [20]. To avoid requiring specialized clinical expertise, the needle insertion path was predefined and automatically guided. A laser-based navigation aid, inspired by commercial systems (e.g., ATLAS), indicated both the puncture site and the insertion angle. Participants aligned the needle with the laser guidance and advanced it to a predefined depth, indicated by a stopper on the needle shaft and applied consistently across all conditions.

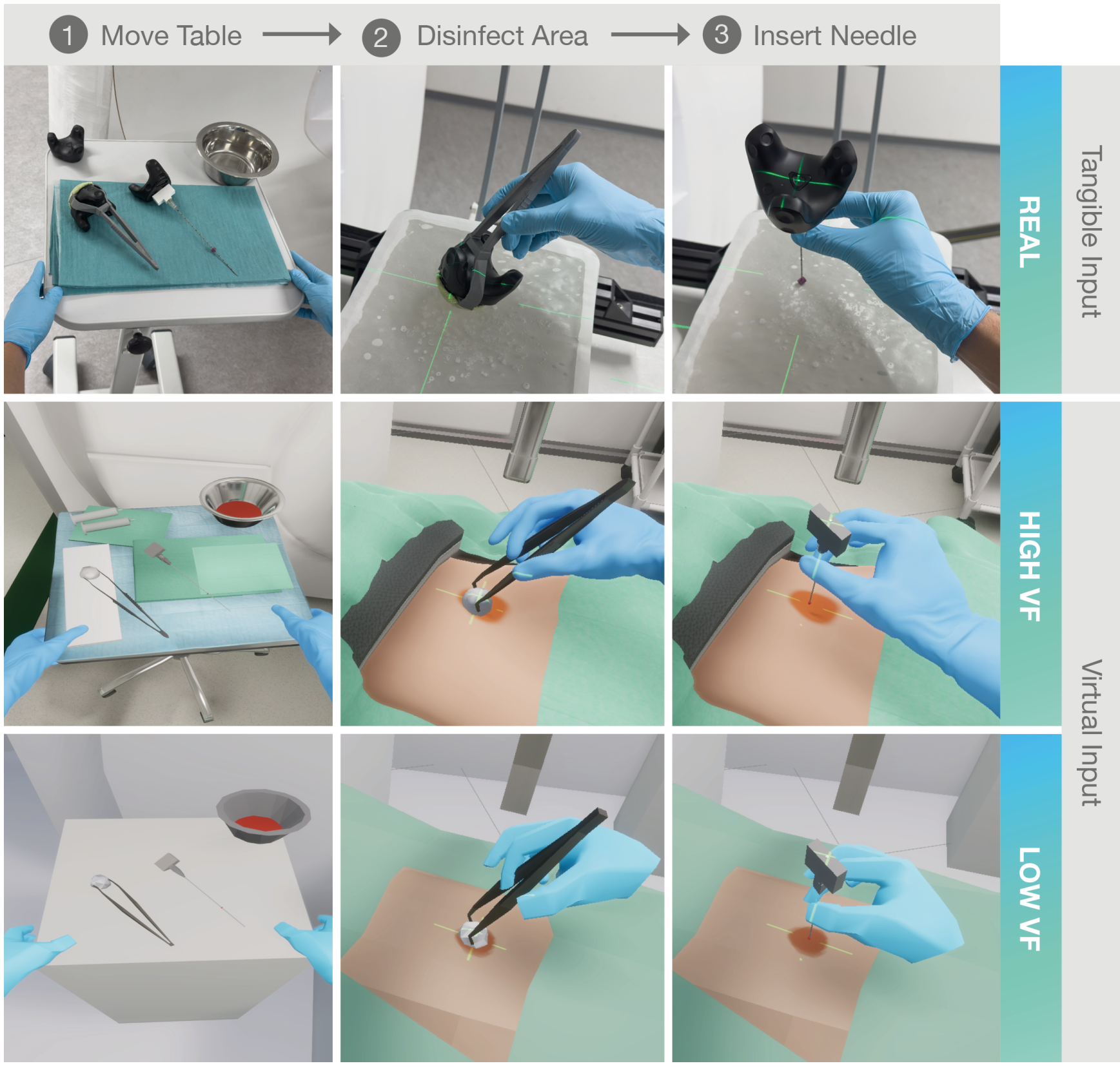

Fig. 1: Overview of the three simulation sub-tasks (left to right): instrument table placement, disinfection of the laser-marked puncture site, and guided needle insertion at a predefined angle and depth. The top row shows tangible tool interaction, including the non-immersive baseline, while the lower rows illustrate virtual interaction across the VF conditions.

2.2 Fidelity Conditions

In line with the definitions introduced above, VF was manipulated through the visual detail and realism of the simulated environment and task-relevant objects, whereas IF was manipulated through the physical correspondence between users' actions, the interaction medium, and the resulting system response. Based on prior work showing that the strongest perceptual differences are observed between low and high levels of VF [3], we contrasted these two conditions:

- **High VF:** High-polygon models, realistic textures, dynamic lighting, and full furnishing to create a visually rich and immersive environment.
- **Low VF:** Simplified geometry, monochrome surfaces, minimal shading, and the absence of props to reflect a low-effort prototyping aesthetic.

Previous work reported no significant effects of interaction modality on UX or performance, potentially due to the similarity of the tested modalities (VR controllers vs. tangible props), both providing tactile input [3]. To better differentiate IF, we therefore contrasted two more distinct interaction conditions:

- **Tangible Input:** Physical tools and props integrated into the IVE via tracked objects, enabling direct hand–object interaction.
- **Virtual Input:** Fully virtual objects manipulated via mid-air hand interaction.

Hand tracking was used in both conditions, with users represented by virtual gloved hands to ensure visual consistency and alignment with medical practice. An overview of the VF and IF conditions is shown in Figure 2 and Figure 1.

2.3 Simulation Environment and Interaction

The simulation was implemented as a hybrid environment combining virtual content with tracked physical components to support tool-based interaction. The IVE replicates an interventional workspace centered around a physical MRI mock-up and includes tracked medical instruments (e.g., table, needle) synchronized in 6DoF with their virtual counterparts. The spatial correspondence between the simulated and physical environment was realized by means of physical measurements and manual registration. A fixed tracker on the MRI mock-up was used as world anchor to save the derived registration.

For tangible input conditions, a candle gel phantom was aligned with the virtual patient anatomy to provide a physical puncture surface. Needle insertion was supported by a tracked laser guidance unit, inspired by commercial systems (e.g., ATLAS), indicating puncture site and insertion angle. In addition to the MR conditions, a non-immersive baseline was implemented in which the same tasks were performed in the physical setup without an HMD, using identical instruments. This condition serves as a real-world reference for comparing performance and subjective measures across immersive and non-immersive settings. Visual comparisons of all setups are shown in Figure 2 and Figure 1.

In the virtual interaction condition, needle insertion was simulated using a simplified physics-based model of tissue resistance. Surface contact with the virtual patient model and insertion depth were detected via raycasting. Upon contact, the system registered puncture initiation and recorded the initial insertion trajectory. Beyond a depth of 4 cm, depth-dependent constraints progressively limited lateral and angular deviation of the needle from this trajectory, approximating the increasing resistance of deeper tissue layers. Exceeding these limits triggered a restoring “rubber band” effect that pulled the needle tip back toward the valid range. This constraint was visualized by a visible offset between the user’s hand position and the constrained needle pose, providing feedback while keeping the model computationally simple.

2.4 System Implementation

The system was implemented using a head-mounted MR setup with combined hand and object tracking. Participants wore a Valve Index HMD equipped with an Ultraleap Leap Motion Controller 2 for markerless optical hand tracking. Tangible instruments, including the needle and instrument table, were tracked in 6DoF using HTC Vive Trackers 3.0 and spatially registered with their virtual counterparts.

The application was developed in Unity 2022.3.16f1 using the High Definition Render Pipeline (HDRP 14.0.9), which supported the implementation of the high- and low-VF conditions through different geometry, material, texture, and lighting configurations. XR integration was realized using the OpenVR XR Plugin and SteamVR Unity Plugin, while hand tracking was integrated using the Ultraleap Unity Plugin. The Ultraleap tracking system used Tracking Service version 5.17.1.

The simulation ran on a Windows 11 Pro workstation equipped with an AMD Ryzen 9 5950X processor, an NVIDIA GeForce RTX 3090 GPU, and 64 GB RAM. Additional third-party tools included the Projector Simulator[1] and TexturePainter[2]. Virtual interaction was visualized using realistic gloved hand models[3].

3 Evaluation

We conducted a within-subjects design user study to investigate the effects of the implemented VF and IF levels. This 2×2 comparison was extended to include the non-immersive baseline condition.

3.1 Sample Design

The tasks did not require professional expertise. However, to ensure basic medical knowledge and familiarity with clinical environments, participants were recruited exclusively from the human medicine program of our institution. Thirty-one participants took part in the study (21 female), providing a statistical power of 0.91 ($\alpha = 0.05$, $\beta = 0.8$, $f = 0.25$). Participants were aged 19–33 years ($Med = 25$) and ranged from the 1st to

[1] https://assetstore.unity.com/packages/tools/particles-effects/projector-simulator-86123
[2] https://github.com/CodeArtistMX/RealTimePainting
[3] https://sketchfab.com/3d-models/rubber-med-gloves-free-to-download-ef128b0efbb1461c8c0f37b83b5f17af

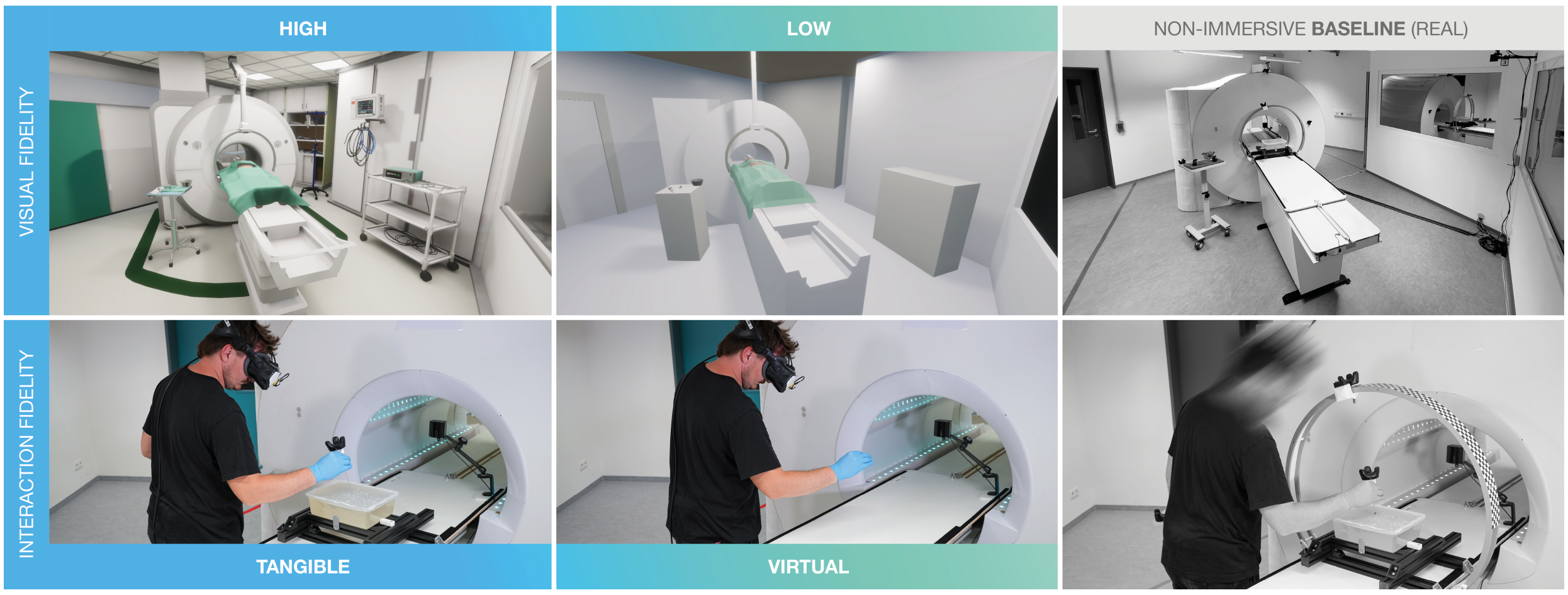

Fig. 2: Overview of the study design. Top row shows the digital twin under high and low visual fidelity and the non-immersive baseline. Bottom row illustrates needle insertion using tangible and virtual interaction, as well as the real-world baseline.

the 17th semester of medical studies ($Med = 10$). All reported normal or corrected-to-normal vision. Self-assessments on 5-point Likert scales indicated medium technical affinity (median 3) and low prior experience with video games and VR (median 2).

3.2 Hypotheses

The study addressed several hypotheses concerning the effects of VF and IF. First, we considered potential interaction effects between them ($H1$). Although VF and IF target different components of the interaction loop, their effects may interact because visual scene information, action execution, and system responses jointly shape task performance and UX. In addition, matching effects between fidelity-related dimensions have been observed in prior VR research [15]. However, due to the lack of prior evidence in precision-critical medical tasks, this hypothesis was examined in an exploratory manner.

Regarding performance, we hypothesized no differences in task completion time or accuracy between high and low VF ($H2.1$), consistent with prior findings in medical MR [3]. In contrast, tangible IF was expected to result in shorter task durations and better accuracy outcomes compared to virtual IF ($H2.2$), based on evidence that tangible input can improve task performance [21].

With respect to subjective measures, we hypothesized that high VF would lead to higher perceived workload compared to low VF, while simultaneously improving UX ($H3.1$), in line with prior work on visual complexity and engagement [3, 11, 22]. Furthermore, we hypothesized that tangible IF would reduce perceived workload and yield higher UX ratings than virtual IF ($H3.2$), as the absence of tactile feedback in virtual-only interaction may increase physical and mental effort and reduce perceived realism [23].

Finally, we hypothesized that high VF would result in higher presence ratings compared to low VF ($H4.1$), reflecting the association between visual realism and immersive experience reported in prior studies [3, 8, 9]. In addition, tangible IF was expected to further increase presence relative to virtual IF ($H4.2$), as physical interaction has been shown to enhance realism and embodiment in immersive simulations [24].

3.3 Variables

Based on the hypotheses above, several dependent variables were measured. Performance analyses focused on sub-task (c), the precision-critical needle insertion. Task completion time (TCT) was measured from needle pickup to an acoustic stop signal, at which point the final needle pose was recorded. Needle accuracy was decomposed into positioning error (distance between the needle–skin intersection and the planned entry point), alignment error (distance between the needle handle marker and the projected guidance laser), and depth error (difference between actual and planned insertion depth). These metrics were chosen to distinguish individual stages of the insertion process. Due to minor registration drift between the tracked and virtual needle in the tangible condition and the absence of a recorded initial contact point, positioning error was excluded from subsequent analyses.

Subjective measures were assessed using standardized questionnaires. Workload was measured with the raw NASA TLX , UX with the short User Experience Questionnaire (UEQ) , and presence with the igroup Presence Questionnaire (IPQ) , including the sub-scales general presence, spatial presence, involvement, and perceived realism.

3.4 Ethical Approval

This study was approved by the university ethics board (ID 119/24). The procedures used in this study adhere to the tenets of the Declaration of Helsinki. Informed consent was obtained from all individual participants included in the study.

3.5 Procedure

Participants completed the simulation individually. After consent, demographics, and a brief introduction, condition order was counterbalanced using a double Latin square design. Each session lasted about one hour and was supervised by two experimenters, one monitoring the application from the control room and one ensuring safe needle handling. In each condition, participants performed the three sub-tasks described in subsection 2.1 in a fixed order: instrument table placement, disinfection of the puncture site, and guided needle insertion. For needle insertion, participants aligned the needle with the laser-guided entry point and angle and inserted it to a predefined depth. Insertion angles (10°–25°) and depths (4.5–7.5 cm) were randomized across trials. In the non-immersive condition, angle and depth were adjusted by the experimenter. Task completion was indicated verbally by the participant and logged by the experimenter. Each condition was repeated four times. The first run served as training and the remaining three were used for analyses. After each condition, participants completed standardized questionnaires (subsection 3.3). This procedure was repeated for all condition combinations and the non-immersive baseline, followed by a final questionnaire.

3.6 Data Processing & Statistical Analyses

Data were analyzed using RStudio. For each condition, performance measures were averaged across repetitions. Questionnaire data were processed to obtain raw NASA TLX scores, overall UEQ scores, and individual IPQ scores. No outliers were removed. Statistical analyses were conducted on the resulting 2×2 factorial data; the non-immersive baseline was analyzed descriptively and included in post-hoc comparisons. Normality and homogeneity of variance were assessed using Shapiro–Wilk and Levene's tests. Variables meeting these assumptions (subjective workload and UX) were analyzed using two-way repeated-measures ANOVAs. For all other measures, robust two-way within-subject ANOVAs based on trimmed means were applied. Non-significant main effects were further examined using Bayesian paired t-tests to assess evidence for the null hypothesis. The ordinal general presence item of the IPQ was analyzed using aligned rank transformation followed by Type III Wald F tests with participant ID as a random effect. Standard ANOVAs report F-statistics and η^2 effect sizes, while robust ANOVAs report Q-statistics and effect sizes δ_t for main effects; η^2 was used for interaction effects.

4 Results

This section describes the statistical results of the experiment in terms of performance, workload, UX and presence. Regarding the final interview, results are summarized in Figure 3 k-l. Most participants preferred high VF and tangible IF. Furthermore, 90% reported that IF had a noticeable impact on task performance, compared to 68% for VF.

4.1 Performance

For needle insertion TCT, a significant VF×IF interaction was observed ($Q = 4.95$, $p = 0.026$, $\eta^2 = 0.008$; Figure 3 a). Pairwise tests indicated that the performance advantage of tangible IF was larger under low VF, while the overall trend remained consistent across VF levels. Given the negligible effect size, main effects were examined. No VF effect was found ($Q = 2.25$, $p = 0.134$, $\delta_t = 0.182$), with moderate evidence supporting the null hypothesis ($BF_{10} = 0.225$). In contrast, IF had a significant effect, with tangible interaction yielding shorter TCT than virtual interaction ($Q = 10.14$, $p = 0.001$, $\delta_t = -0.475$). A similar pattern emerged for needle alignment error. A significant but small interaction effect was found ($Q = 5.87$, $p = 0.016$, $\eta^2 = 0.006$; Figure 3 b), with tangible IF producing lower errors across both VF levels and a larger effect under low VF. No main effect of VF was observed ($Q = 1.87$, $p = 0.172$, $\delta_t = -0.143$; $BF_{10} = 0.235$), whereas IF showed a strong main effect favoring tangible interaction ($Q = 54.08$, $p < 0.001$, $\delta_t = -1.201$). For depth error, significant main effects of VF ($Q = 7.40$, $p = 0.007$, $\delta_t = -0.370$) and IF ($Q = 10.43$, $p = 0.001$, $\delta_t = -0.489$) were found, with no interaction effect ($Q = 0.94$, $p = 0.333$). Both high VF and tangible IF resulted in more accurate depth control (Figure 3 c-d). It is notable that both VF conditions showed no difference in the post-hoc tests when compared with the non-immersive baseline condition.

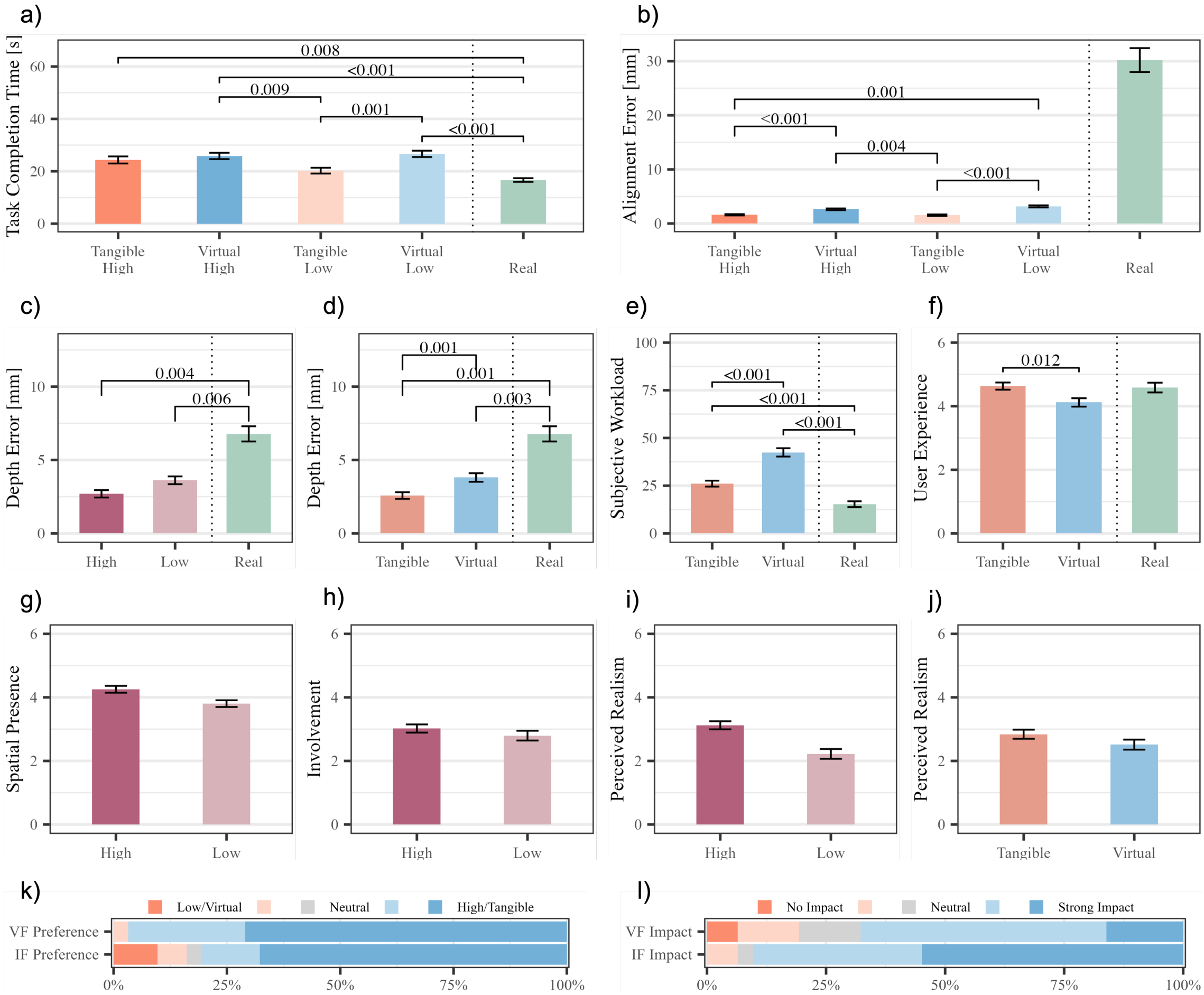

Fig. 3: Overview of experimental outcomes. (a) Significant interaction effects on TCT. (b) Significant interaction effects on alignment error (Real is also significantly different from all other conditions). (c) VF main effect on depth error. (d) IF main effect on depth error. (e) IF main effect on subjective workload. (f) IF main effect on UX. (g) VF main effect on spatial presence. (h) VF main effect on involvement. (i) VF main effect on perceived realism. (j) IF main effect on perceived realism. (k) Final interview results showing VF and IF preference. (l) Final interview results showing perceived impact ratings.

4.2 Workload and UX

For subjective workload, no significant VF main effect was observed ($Q = 0.64$, $p = 0.425$, $\delta_t = -0.020$), with moderate evidence supporting the null hypothesis ($BF_{10} = 0.218$). In contrast, a significant IF main effect was found, with tangible interaction resulting in lower workload than virtual interaction ($Q = 20.79$, $p < 0.001$, $\delta_t = -0.898$; Figure 3 e). No interaction effect occurred ($Q = 0.22$, $p = 0.642$, $\eta^2 < 0.001$). Similarly, UX ratings showed no significant VF effect ($Q = 2.05$, $p = 0.153$, $\delta_t = 0.150$), supported by anecdotal evidence for the null hypothesis ($BF_{10} = 0.489$), but a significant IF main effect favoring tangible interaction ($Q = 6.49$, $p = 0.011$, $\delta_t = 0.434$; Figure 3 f), with no interaction ($Q = 0.03$, $p = 0.855$, $\eta^2 < 0.001$).

4.3 Presence

For general presence, no significant VF ($F = 3.83$, $p = 0.054$, $BF_{10} = 0.345$) or IF main effect ($F = 0.20$, $p = 0.654$, $BF_{10} = 0.155$) was found, and no interaction occurred ($F = 1.75$, $p = 0.287$). Bayes factor analyses indicated anecdotal evidence for the VF null hypothesis and moderate evidence for the IF null hypothesis. In contrast, spatial presence showed a significant VF main effect, with high VF yielding higher ratings ($F = 20.56$, $p < 0.001$, $\eta^2 = 0.062$; Figure 3 g), while IF had no effect ($F = 0.77$, $p = 0.386$, $\eta^2 = 0.005$), supported by moderate evidence for the null hypothesis ($BF_{10} = 0.226$). No interaction was observed ($F = 1.91$, $p = 0.178$, $\eta^2 = 0.006$).

A similar pattern was found for involvement, with a significant VF main effect favoring high VF ($F = 4.80$, $p = 0.037$, $\eta^2 = 0.011$; Figure 3 h), no IF effect ($F = 0.35$, $p = 0.561$, $\eta^2 = 0.001$), and moderate evidence supporting the IF null hypothesis ($BF_{10} = 0.176$). No interaction occurred ($F = 0.08$, $p = 0.775$, $\eta^2 < 0.001$). Finally, perceived realism showed significant main effects of both VF ($F = 37.83$, $p < 0.001$,

$\eta^2 = 0.146$) and IF ($F = 4.74$, $p = 0.038$, $\eta^2 = 0.026$; Figure 3 i-j), with no interaction ($F = 0.43$, $p = 0.519$, $\eta^2 = 0.001$).

5 Discussion

Both VF and IF factors successfully manipulated realism, as reflected by significant main effects on perceived realism, confirming the intended fidelity manipulation and aligning with prior work [3, 23]. With respect to $H1$, small but statistically significant VF×IF interaction effects were observed for TCT and alignment error. However, these effects were below the threshold for small effect sizes and did not alter the ordinal relations between conditions. Consequently, interaction effects should be interpreted with caution and primarily as an indication that fidelity dimensions may not act independently in all contexts.

Regarding VF, participants expressed a clear preference for high VF and reported a perceived impact on performance. Objectively, however, VF had no effect on TCT or alignment error, supported by moderate Bayesian evidence for the null hypothesis, thereby supporting $H2.1$. In contrast, depth accuracy was significantly improved under high VF, indicating that enhanced lighting and shading may support depth perception in fine motor tasks. Furthermore, high VF increased spatial presence, involvement, and perceived realism, confirming $H4.1$ and replicating prior findings [8, 9]. No VF effects were found for workload or UX, leading to a rejection of $H3.1$.

IF showed stronger and more consistent effects across measures. Tangible input resulted in shorter TCT, lower alignment and depth errors, reduced workload, and higher UX, thereby supporting both $H2.2$ and $H3.2$. These findings are consistent with prior work highlighting the benefits of tactile feedback for precision and usability [21]. In contrast, IF had no effect on general presence, spatial presence, or involvement, with Bayesian analyses supporting the respective null hypotheses. Consequently, $H4.2$ must be rejected, indicating that presence in precision-critical MR tasks is more strongly influenced by VF than by interaction modality.

The non-immersive baseline condition provided additional context. Due to registration limitations, accuracy measures could not be meaningfully compared. However, TCT, workload, and UX indicated that tangible MR interaction most closely approximated real-world performance, while the baseline produced the lowest workload, likely due to the absence of headset-related cognitive and physical demands [25]. These findings further underline the importance of IF over VF when approximating real-world task execution in MR.

This study was not designed to assess skill transfer directly, but rather to disentangle how VF and IF affect core components of simulation quality. The observed dissociation—performance benefits driven primarily by IF and experiential benefits driven primarily by VF—provides a structured foundation for future transfer studies. Several limitations remain, including tracking inaccuracies, ergonomic differences of the tracked needle, visual clipping artifacts, registration errors affecting positioning accuracy, the use of a student sample, and residual tactile cues in the virtual condition. Manual registration between physical and virtual components may have contributed to residual spatial misalignment. In addition, the virtual insertion condition used a simplified approximation of tissue resistance, limiting direct transferability to clinical procedures. Addressing these issues in future work will be essential for evaluating training transfer and clinical applicability.

6 Conclusion

In this work, we examined the effects of VF and IF on performance and experience in a precision-critical medical MR simulation of a guided needle insertion task. While both fidelity dimensions successfully increased perceived realism, their functional roles differed markedly. IF had a consistent and substantial impact on procedural performance, reducing task completion time, alignment and depth errors, and subjective workload, whereas VF showed only limited performance effects but reliably increased spatial presence, involvement, and perceived realism. Importantly, increases in presence and UX did not necessarily translate into improved performance, indicating that experiential and procedural outcomes can be decoupled in high-precision medical MR tasks. From a medical simulation perspective, these findings suggest that accurate and tangible interaction is a primary determinant of effective task execution and should be prioritized when designing MR systems for performance evaluation, training, or workflow prototyping. VF, while less critical for accuracy, remains relevant for immersive engagement and perceived realism and should be selectively increased depending on training goals. Overall, this work provides empirical guidance for aligning fidelity choices in MR-based medical simulation with the intended clinical, educational, or evaluative purpose of the system.

7 Declaration of competing interest

The authors declare that they have no known competing financial interests or personal relationships that could have influenced the work reported in this paper.

8 Acknowledgments

This work was carried out as part of the STIMULATE research campus (German Federal Ministry of Research, Technology, and Space grant number 13GW0473A, and European Regional Development Fund grant number ZS/2023/12/182010).

References

[1] Tene, T., Vique López, D.F., Valverde Aguirre, P.E., Orna Puente, L.M., Vacacela Gomez, C.: Virtual reality and augmented reality in medical education: An umbrella review. Front. Digit. Health **6**, 1365345 (2024) https://doi.org/10.3389/fdgth.2024.1365345

[2] Sánchez-Margallo, J.A., Miguel, C., Fernández Anzules, R.A., Sánchez-Margallo, F.M.: Application of mixed reality in medical training and surgical planning focused on minimally invasive surgery. Front. Virtual Real. **2**, 692641 (2021) https://doi.org/10.3389/frvir.2021.692641

[3] Schott, D., Heinrich, F., Stallmeister, L., Moritz, J., Hensen, B., Hansen, C.: Is this the vreal life? manipulating visual fidelity of immersive environments for medical task simulation. In: Proc. IEEE Int. Symp. Mixed Augment. Reality (ISMAR), pp. 1171–1180 (2023). https://doi.org/10.1109/ISMAR59233.2023.00134

[4] Zhou, X., Rau, P.-L.P.: Determining fidelity of mixed prototypes: Effect of media and physical interaction. Appl. Ergon. **80**, 111–118 (2019) https://doi.org/10.1016/j.apergo.2019.05.007

[5] Jung, S., Lindeman, R.W.: Perspective: Does realism improve presence in VR? suggesting a model and metric for VR experience evaluation. Front. Virtual Real. **2**, 693327 (2021) https://doi.org/10.3389/frvir.2021.693327

[6] Slater, M., Wilbur, S.: A framework for immersive virtual environments (five): Speculations on the role of presence in virtual environments. Presence: Teleoperators & Virtual Environments **6**(6), 603–616 (1997)

[7] Melo, M., Gonçalves, G., Monteiro, P., Coelho, H., Vasconcelos-Raposo, J., Bessa, M.: Do multisensory stimuli benefit the virtual reality experience? a systematic review. IEEE Trans. Vis. Comput. Graph. **28**(2), 1428–1442 (2022) https://doi.org/10.1109/TVCG.2020.3010088

[8] Hvass, J., Larsen, O., Vendelbo, K., Nilsson, N., Nordahl, R., Serafin, S.: Visual realism and presence in a virtual reality game. In: 2017 3DTV Conf.: The True Vision – Capture, Transmission and Display of 3D Video (3DTV-CON), pp. 1–4. IEEE, Copenhagen, Denmark (2017). https://doi.org/10.1109/3DTV.2017.8280421

[9] Newman, M., Gatersleben, B., Wyles, K.J., Ratcliffe, E.: The use of virtual reality in environment experiences and the importance of realism. J. Environ. Psychol. **79**, 101733 (2022) https://doi.org/10.1016/j.jenvp.2021.101733

[10] Bonfert, M., Muender, T., McMahan, R.P., Steinicke, F., Bowman, D., Malaka, R., Döring, T.: The interaction fidelity model: A taxonomy to communicate the different aspects of fidelity in virtual reality. Int. J. Hum.-Comput. Interact. **41**(12), 7593–7625 (2024) https://doi.org/10.1080/10447318.2024.2400377

[11] Gonçalves, G., Coelho, H., Monteiro, P., Melo, M., Bessa, M.: Systematic review of comparative studies of the impact of realism in immersive virtual experiences. ACM Comput. Surv. **55**(6), 1–36 (2022) https://doi.org/10.1145/3533377

[12] Roh, Y.S., Jang, K.I., Lee, E.A.L.: Influencing factors and learning outcomes in virtual simulation learning experiences: A structural equation modeling. Nurse Educ. Today **150**, 106681 (2025) https://doi.org/10.1016/j.nedt.2025.106681

[13] Harman, J., Brown, R., Johnson, D., Turkay, S.: The role of visual detail during situated memory recall within a virtual reality environment (2019) https://doi.org/10.1145/3369457.3369469

[14] Schmied-Kowarzik, R.L., Paelke, V.: Examining the importance of realism in virtual reality therapy environments for people with specific phobias. In: GI VR/AR Workshop. Gesellschaft für Informatik, Bonn, Germany (2021). https://doi.org/10.18420/vrar2021_16

[15] McMahan, R.P., Bowman, D.A., Zielinski, D.J., Brady, R.B.: Evaluating display fidelity and interaction fidelity in a virtual reality game. IEEE Trans. Vis. Comput. Graph. **18**(4), 626–633 (2012) https://doi.org/10.1109/TVCG.2012.43

[16] Rodrigues, F., Giovannelli, A., Pavanatto, L., Miao, H., Oliveira, J.C.d., Bowman, D.A.: AMP-IT and WISDOM: Improving 3d manipulation for high-precision tasks in virtual reality. In: Proc. IEEE Int. Symp. Mixed Augment. Reality (ISMAR), Sydney, Australia, pp. 303–311 (2023). https://doi.org/10.1109/ISMAR59233.2023.00045

[17] Rzepka, A.M., Hussey, K.J., Maltz, M.V., Babin, K., Wilcox, L.M., Culham, J.C.: Familiar size affects perception differently in virtual reality and the real world. Philos. Trans. R. Soc. B **378**(1869) (2022) https://doi.org/10.1098/rstb.2021.0464

[18] Persky, S., Jiao, M.G.: Extended reality analgesia evidence reviews often lack sufficient intervention detail. J. Med. Ext. Real. **1**(1), 290–305 (2024) https://doi.org/10.1089/jmxr.2024.0043

[19] Plümer, J.H., Yu, K., Eck, U., Kalkofen, D., Steininger, P., Navab, N., Tatzgern, M.: XR Prototyping of Mixed Reality Visualizations: Compensating Interaction Latency for a Medical Imaging Robot. arXiv preprint. arXiv:2409.xxxxx (2024)

[20] Shan, T., Wang, K., Feng, Y., Huang, J., Jiang, H.: Efficacy and influencing factors of the four-step approach combining the situational simulation teaching method in the clinical practice of standardized training for residents. Health Sci. Rep. **5**(5), 757 (2022) https://doi.org/10.1002/hsr2.757

[21] Kourtesis, P., Vizcay, S., Marchal, M., Pacchierotti, C., Argelaguet, F.: Action-specific perception and performance on a Fitts's law task in virtual reality: The role of haptic feedback. IEEE Trans. Vis. Comput. Graph. **28**(11), 3715–3726 (2022) https://doi.org/10.1109/TVCG.2022.3203003

[22] Kleinbeck, C., Zhang, H., Killeen, B.D., Roth, D., Unberath, M.: Neural digital twins: Reconstructing complex medical environments for spatial planning in virtual reality. Int. J. Comput. Assist. Radiol. Surg. **19**(7), 1301–1312 (2024) https://doi.org/10.1007/s11548-024-03143-w

[23] Rettinger, M., Rigoll, G.: Touching the future of training: Investigating tangible interaction in virtual reality. Front. Virtual Real. **4** (2023) https://doi.org/10.3389/frvir.2023.1187883

[24] Muender, T., Reinschluessel, A.V., Drewes, S., Wenig, D., Döring, T., Malaka, R.: Does it feel real?: Using tangibles with different fidelities to build and explore scenes in virtual reality. In: Proc. 2019 CHI Conf. Hum. Factors Comput. Syst., pp. 1–12. ACM, Glasgow, UK (2019). https://doi.org/10.1145/3290605.3300903

[25] Frederiksen, J.G., Sørensen, S.M.D., Konge, L., Svendsen, M.B.S., Nobel-Jørgensen, M., Bjerrum, F., Andersen, S.A.W.: Cognitive load and performance in immersive virtual reality versus conventional virtual reality simulation training of laparoscopic surgery: A randomized trial. Surg. Endosc. **34**(3), 1244–1252 (2019) https://doi.org/10.1007/s00464-019-06887-8
